# Recent Advances on the Neuroprotective Potential of Antioxidants in Experimental Models of Parkinson’s Disease

**DOI:** 10.3390/ijms130810608

**Published:** 2012-08-23

**Authors:** Sushruta Koppula, Hemant Kumar, Sandeep Vasant More, Byung Wook Kim, In Su Kim, Dong Kug Choi

**Affiliations:** Department of Biotechnology, Konkuk University, Chungju 380, Korea; E-Mails: sushrutak@gmail.com (S.K.); hemantbhave@gmail.com (H.K.); sandeepbcp@gmail.com (S.V.M.); kb62@lycos.co.kr (B.W.K.); kis5497@hanmail.net (I.S.K.)

**Keywords:** Parkinson’s disease, oxidative stress, free radicals, antioxidants, neuroprotection

## Abstract

Parkinson’s disease (PD), a neurodegenerative movement disorder of the central nervous system (CNS) is characterized by a progressive loss of dopaminergic neurons in the *substantia nigra pars compacta* region of the midbrain. Although the etiology of PD is not completely understood and is believed to be multifactorial, oxidative stress and mitochondrial dysfunction are widely considered major consequences, which provide important clues to the disease mechanisms. Studies have explored the role of free radicals and oxidative stress that contributes to the cascade of events leading to dopamine cell degeneration in PD. In general, in-built protective mechanisms consisting of enzymatic and non-enzymatic antioxidants in the CNS play decisive roles in preventing neuronal cell loss due to free radicals. But the ability to produce these antioxidants decreases with aging. Therefore, antioxidant therapy alone or in combination with current treatment methods may represent an attractive strategy for treating or preventing the neurodegeneration seen in PD. Here we summarize the recent discoveries of potential antioxidant compounds for modulating free radical mediated oxidative stress leading to neurotoxicity in PD.

## 1. Introduction

Parkinson’s disease (PD) is a progressive and age-related neurodegenerative disease characterized by degeneration of dopaminergic (DAergic) neurons originating in the *substantia nigra pars compacta* (SNpc) of the basal ganglia [1,2]. Despite major advances in the current understanding of PD pathology, the exact details of the neurodegenerative cascade remain unknown. Experimental observations suggest that excessive generation of reactive oxygen species (ROS), which cause oxidative stress, plays a central role in the neuropathology of PD. This theory of oxidative stress is supported by both postmortem studies and by studies demonstrating the capacity of oxidative stress and oxidizing toxins to nigral cell degeneration [3,4]. Excessive formation of ROS leading to increased lipid peroxidation [5], oxidative damage of DNA [6], glutathione (GSH) depletion [7], enhanced superoxide activity [8], increased levels of iron [9] and subsequent cellular apoptosis are considered leading factors in the oxidative metabolism of DA observed in PD pathology. Mitochondrial dysfunction by ROS can also give rise to DAergic neurodegeneration [10], as DNA is particularly sensitive to hydroxyl radical-induced damage.

Treatment of PD with the drug of choice, l-dopa, is limited only to the relief of symptoms, and long-term use may further add to the oxidative load by producing free radicals during normal metabolism and play a role in disease progression [11–17]. Although other classes of drugs such as DA agonists, monoamine oxidase (MAO) inhibitors, catechol-O-methyltransferase inhibitors, and anticholinergic agents may be used in the early stages of the disease to relieve PD symptoms, none prevent the disease from progressing, and show debilitating side-effects with prolonged use. Therefore, it is of utmost importance to develop new agents that show or halt the rate of PD progression. The key therapy to ameliorate oxidative stress seen in PD is to repair the damage caused by free radicals before it is too late and to protect DAergic cells. Therefore, antioxidants might be one of the ideal agents to prevent free radical-mediated tissue destruction and inhibit some of the early degenerative events trafficking in the central nervous system that lead to neurodegeneration in PD and its experimental models.

The protective effects of various antioxidants to modulate oxidative stress in experimental animal models of PD have been clearly shown, indicating that antioxidant therapy may be an attractive therapeutic approach to PD. The source of ROS production and evidence for ROS and oxidative stress in PD has been reviewed extensively [18,19]. In this review, we provide recent discoveries of neuroprotective antioxidant compounds as a therapeutic strategy for inhibiting free radicals and oxidative damage in experimental models of PD.

## 2. PD and ROS

Normal cellular functions and reactions involve the continuous production of free radicals, mainly ROS. Some ROS, such as superoxide anion, nitric oxide, and hydrogen peroxide (H_2_O_2_) are physiological species essential for redox signaling and cellular functions. In-built protective mechanisms consisting of enzymatic and non-enzymatic antioxidants scavenge these radicals. However, uncontrolled production of ROS may threaten homeostasis, as the required endogenous antioxidants are insufficient and may decrease with aging. This imbalance may lead to excessive production of non-physiological and toxic ROS levels in a process referred to as oxidative stress.

The brain’s neuronal biochemical composition is mainly susceptible to ROS, as it involves a pool of unsaturated lipids that are labile to peroxidation and oxidative modification. Furthermore, the brain is not particularly enriched in antioxidant defenses compared to those in other tissues [20]. Irregular cellular functions in the brain may produce enormous concentrations of ROS that promote the neuronal damage seen in PD [21]. The premise of this hypothesis is based on landmark studies demonstrating the potential for generating H_2_O_2_ and other ROS during the oxidative metabolism of DA [11], which exposes DAergic neurons of the SNpc to chronic oxidative stress compared to other regions of the brain. Other major factors responsible for non-physiological ROS production and their importance in PD are transitional metals, including iron [22,23], mitochondrial abnormalities [10,24,25], alpha-synuclein (SNCA) gene expression [26–28], inflammation mediated by microglial activation [29], reduced levels of endogenous antioxidant nutrients (glutathione [GSH] and ascorbic acid), and antioxidant enzymes (catalase [CAT] and GSH peroxidase).

To study the role of free radical damage in PD, it is essential to develop suitable animal models that can be used for the screening and testing of new therapeutic strategies targeting the actual pathogenic process as opposed to merely developing symptomatic therapies. Several PD models, including genetic and toxin-induced models, have been developed to understand the intrinsic mechanisms to gain insight into PD pathogenesis. *In vitro* utilization of various toxin-induced cell lines such as PC12, SH-SY5Y, and MN9D mimic many aspects of DAergic neuron death observed in PD. The *in vivo* classical animal models of PD rely on the systemic or intracerebral administration of neurotoxins such as reserpine, haloperidol, 1-methyl 4-phenyl-1,2,3,6-tetrahydropyridine (MPTP), 6-OHDA, and, more recently, the pesticides rotenone, maneb, and paraquat, which have the ability to generate ROS in neurons and induce oxidative damage in the nigrostriatal DAergic system [1,30–37]. Several genes associated with PD, including SNCA, parkin, DJ-1, PINK1, and LRRK2 are linked with mitochondrial dysfunction and oxidative stress [38,39]. Although genetics has generated tremendous excitement and new energy in PD research, it is important to realize that only 10%–20% of PD is due to genetic causes [40]. These critical data provide us with immense evidence that ROS and oxidative stress play a major role in the development of PD.

## 3. Role of Neuroprotective Antioxidant Compounds and Recent Discoveries in Experimental Models of PD

Antioxidants are widely discussed in both the lay press and the scientific literature as health promoting agents that may protect against various age-related diseases. Antioxidants are exogenous or endogenous molecules that act against any form of oxidative stress and its associated ill effects on cellular systems. The state of oxidative imbalance found during neurodegenerative processes is triggered by one or more factors such as brain aging, genetic predisposition, mitochondrial dysfunction, free radical production, and environmental toxins [41–43]. To overcome free radical-mediated consequences of disease processes and drug therapies, antioxidants are now being looked upon as persuasive therapeutics against neuronal loss, as they have the capability to neutralize free radicals.

Antioxidants could provide a significant therapeutic breakthrough in the treatment of PD. Reports have revealed that neurodegeneration in PD is linked to dietary habits in which a deficiency of antioxidant compounds such as folic acid [44], vitamins (A, C, E, and niacin), and selenium in the body increase the risk for PD [45,46]. The therapeutic approach to PD treatment should include modulation of oxidative stress using antioxidants. In the following sections we discuss the recent antioxidant compounds reported during the last five years that show beneficial effects in neuroprotection and in experimental models of PD.

## 4. Antioxidant Compounds in Experimental Models of PD

**Figure 1:**
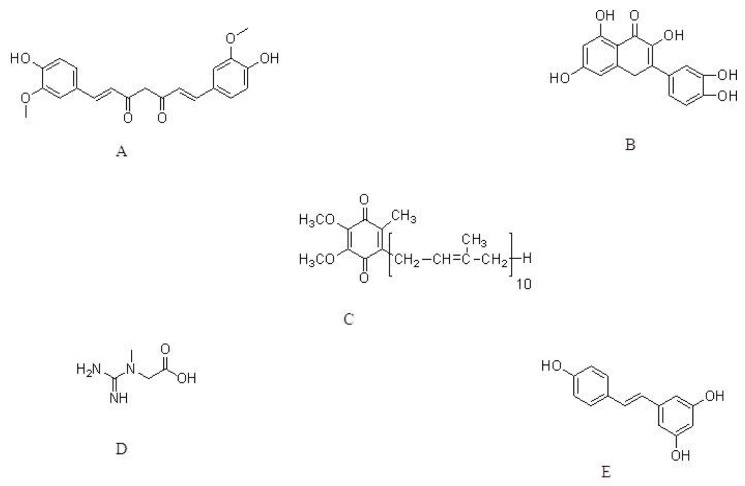
The molecular structure of Curcumin (**A**); Quercetin (**B**); Coenzyme Q10 (**C**); Creatine (**D**) and Resveratrol (**E**).

Curcumin, the well-known component of yellow curry spice (Figure 1A) derived from turmeric, has been used as a food preservative and herbal medicine in India for hundreds of years [47,48]. Curcumin possesses therapeutic properties against a variety of diseases ranging from cancer to cystic fibrosis [49]. Curcumin exhibits antioxidative and antiinflammatory activities [50,51]. Earlier pharmacological studies showed that curcumin has cholesterol-lowering and hemostatic properties. Several studies in cellular and animal models also indicate that curcumin is a neuroprotective agent in neurodegenerative disorders such as Alzheimer’s disease (AD) [52–54] and PD [52,55,56]. In a recent study conducted by Mythri *et al.* (2011) [57], chronic dietary supplementation with turmeric protected against MPTP-mediated neurotoxicity *in vivo* in a mice model of PD. Mice were subjected to dietary supplementation with aqueous suspensions of turmeric for three months, mimicking chronic consumption, and were then challenged *in vivo* with MPTP. The authors showed that chronic turmeric supplementation increased GSH levels and protected against peroxynitrite-mediated inhibition of brain mitochondrial complex I. In another study, Liu *et al.* (2011) explored the protective effects of curcumin against A53T SNCA-induced toxicity in PC12 cells. Those authors showed that curcumin administration (0.1, 0.5, and 1.0 μM) protected against A53T SNCA-induced cell death in a dose-dependent manner by reducing the mutant SNCA-induced intracellular ROS levels, mitochondrial depolarization, cytochrome *c* release, and caspase-9 and caspase-3 activation [58]. The strong antioxidant activity of curcumin makes it an interesting neuroprotective candidate for counteracting the toxin-induced oxidative stress and damage seen in experimental PD models. However the potential risks and side effects of curcumin need to be addressed and seem to be necessary to establish the benefit/risk profile of curcumin as a neuroprotective agent [59].

Quercetin (Figure 1B), a major flavonoid, deserves attention because of its beneficial effects observed in various *in vitro* and *in vivo* neural damage models. Quercetin possesses anti-tumoral, anti-thrombotic, anti-inflammatory, anti-apoptotic, and antioxidant effects [60–63]. In a recent study by Zhang *et al*. (2011) the neuroprotective effects of quercetin in PC12 cells and in a zebrafish model were investigated. 6-OHDA was used to induce neural damage in PC12 cells and zebrafish. Quercetin at 25, 50, and 100 μM prevented 6-OHDA-induced PC12 cell apoptosis. In the zebrafish model, pretreatment with quercetin at 6 and 12 μM significantly attenuated 6-OHDA-stimulated DAergic neuron loss leading to its development as an effective therapeutic agent for treating PD [64]. In addition, new roles for quercetin in hypoxia and ischemia-induced neuroprotection in relation to suppression of oxidative stress, improvement in behavioral function, reduction in infarct volume, brain swelling, and cellular injury in both *in vivo* and *in vitro* models based on its antioxidant functions are also well studied [65]. Although quercetin is regarded as a safe agent, care should be taken when administering in combination with other drugs clinically as it was known to interact with some antibiotics and alter the serum levels [66].

Coenzyme Q10 (CoQ10, Figure 1C) is a key component of the electron transport chain and plays an essential role in ATP production. CoQ10, also called ubiquinone, is absorbed in brain fluids and is a very powerful antioxidant in both mitochondria and lipid membranes [67,68]. Earlier research has shown that the CoQ10 content of mitochondria in the brain declines rapidly when PD is induced in monkeys. This reduction in CoQ10 level leads to a detrimental increase in free radical destructive reactions [69]. CoQ10 possesses neuroprotective properties as observed in different models of neurodegenerative diseases [10,70–72]. CoQ10 also attenuates ATP and GSH depletion and protects against loss of hippocampal neurons in experimental ischemia [73]. CoQ10 significantly decreases lipid peroxidation markers in plasma, erythrocytes, liver, and the brain of mice [74]. In a recent study by Cleren *et al*. (2008) [75] the therapeutic effects of CoQ10 and reduced CoQ10 in the MPTP model of Parkinsonism mice was studied. CoQ10 administered at 1600 mg/kg/day resulted in significant protection against loss of DA induced by MPTP treatment (10 mg/kg, i.p., each 2 h × 3 doses), which was accompanied by a marked increase in plasma concentrations of CoQ10. In a chronic MPTP model (40 mg/kg per day for 1 month), CoQ10 treatment at 1600 mg/kg/day in the diet also showed excellent therapeutic effects by significantly inhibiting striatal DA depletion, loss of dopaminergic neurons in the SNpc, and the formation of SNCA aggregates in the dopaminergic neurons of mice. Results from this study provide further evidence that administering CoQ10 may be a useful therapeutic strategy for treating PD. Because of the paucity of side-effects seen with CoQ10, this powerful antioxidant is now under clinical trial [76].

CoQ10 and creatine (Figure 1D) combination therapy has been investigated in a MPTP mouse model of PD [77]. Earlier reports indicated that creatine exerts neuroprotective effects both *in vitro* and *in vivo* in animal models of neurodegenerative diseases [78]. Those authors showed that supplementation with these combined agents in mice through a diet with 2% creatine and 1% CoQ10 for one week before MPTP treatment (40 mg/kg body weight daily for 28 days through osmotic pumps) produced additive neuroprotective effects against dopamine depletion in the striatum and loss of tyrosine hydroxylase (TH) neurons in the SNpc, reduced lipid peroxidation and pathologic SNCA accumulation in SNpc neurons, and loss of DAergic neurons. They suggested that the combination of the two compounds is a promising approach to PD pathology, as clinical trials of both CoQ10 and creatine are promising and well-tolerated with few side-effects.

Resveratrol (Figure 1E) is a well known antioxidant that exerts extensive pharmacological effects including anti-inflammatory, anti-mutation, anti-tumor and blood fat regulatory functions [79–81]. Resveratrol and a resveratrol liposome were evaluated for their neuroprotective effects in PD [82]. The parameters, including behavior, TH-positive cells, apoptotic cells, ROS level, and total antioxidant capacity were determined in 6-OHDA-induced rats. Oral treatment with resveratrol or a resveratrol liposome (20 mg/kg per day) for 14 days protected DAergic neurons in PD rats. The levels of total ROS decreased markedly, and the total antioxidant capability of nigral tissues improved significantly. Furthermore, the resveratrol liposome exerted more potent protection when compared to that of resveratrol. The authors concluded that the radical scavenging ability and antioxidant properties of resveratrol may contribute to its potent neuroprotection in PD. Further, a single dose of up to 5 g of resveratrol caused no serious adverse effects in healthy volunteers in clinical studies owing to its safe use for neuroprotection [83].

**Figure 2:**
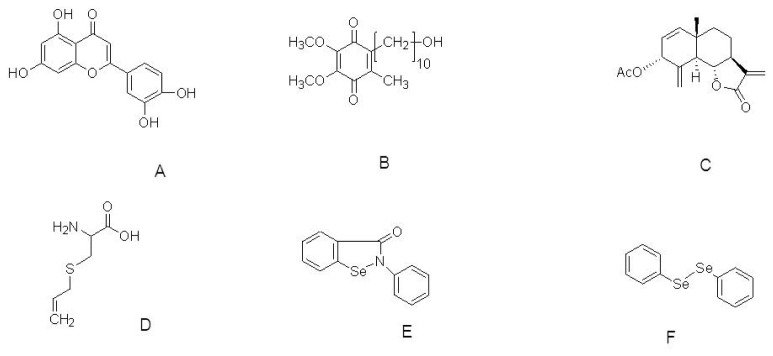
The molecular structure of Luteolin (**A**); Idebenone (**B**); 3α-acetoxyeudesma-1,4(15),11(13)-trien-12,6a-olide (**C**); *S*-Allylcysteine (**D**); Ebselen (**E**) and Diphenyl diselenide (**F**).

Luteolin (Figure 2A) is a polyphenolic compound found in many foods including peanut shells, parsley, artichoke leaves, celery, peppers, olive oil, rosemary, lemons, peppermint, sage, and thyme. This food-derived compound is also one of the dominant active constituents of purple Perilla fruit. Luteolin possesses anti-inflammatory, anti-allergic, anti-carcinogenic, and immune-modulating properties [84]. In a recent study, luteolin derived from *Perilla frutescens* (L) Britt, was investigated for its neuroprotective tendency towards ROS-insulted neural cells. Luteolin concentration-dependently enhances neuronal cell survival with an efficacy higher than and a potency similar to vitamin E when cells were insulted with ROS. Luteolin (5, 10, and 20 μM) significantly attenuated the increase in ROS production and prevented decreases in activities of mitochondria, CAT, and GSH in ROS-insulted primary neurons. That study indicated that the neuroprotection exerted by luteolin in ROS-insulted primary neurons might occur through a rebalancing of pro-oxidant-antioxidant status [85].

Brassinosteroids (BRs) are highly oxygenated steroids isolated from several vegetables, including *Vicia faba* seeds and pollen [86,87]. BRs exert antioxidative actions by enhancing the activity of the enzymatic antioxidant superoxide dismutase (SOD), CAT and peroxidase, to reduce lipid peroxidation [88–90]. In a recent study, two natural BRs and five synthetic analogs were synthesized and evaluated for their neuroprotective actions against MPP^+^-induced neuronal PC12 cells. The authors suggested that selected BRs and analogs protected neuronal PC12 cells against MPP^+^ toxicity and exerted neuroprotective effects derived from their antioxidative properties. In addition, they reported that the steroid B-ring and lateral chain play an important antioxidative role in the neuroprotective action and further research on *in vivo* animal models of PD should be conducted [91].

Gerhardt *et al*. (2011) explored the compound idebenone (Figure 2B) to extend lifespan and improve motor function in HtrA2 knockout mice. Feeding HtrA2 knockout mice with idebenone (500 mg/kg body weight/day orally) extended lifespan and delayed worsening of the motor phenotype. Experiments conducted in cell culture and on brain tissue of mice revealed that idebenone acts by down-regulating the integrated stress response. Earlier reports indicated that idebenone has antioxidant properties similar to CoQ10, which is in use as an anti-aging product based on the free-radical theory [92]. The authors reported that idebenone ameliorates disease symptoms in HtrA2 knockout mice indicating that antioxidants might delay neuronal degeneration in the striata of these mice. This result illustrates the potential of idebenone for treating neurodegenerative diseases including PD [93].

3α-acetoxyeudesma-1,4(15),11(13)-trien-12,6a-olide (AETO, Figure 2C), is a compound isolated from the leaves of *Laurus nobilis* L., The inhibitory effects of AETO on DA-induced apoptosis and SNCA formation in DAergic SH-SY5Y cells have been evaluated. AETO (0.4, 2, and 10 μM) decreases the active form of caspase-3 and the levels of p53, which were accompanied by increased levels of Bcl-2 in a dose-dependent manner. Flow cytometry and Western blot analyses showed that AETO significantly inhibits DA-induced apoptosis and suppresses intracellular tyrosinase activity, ROS generation, quinoprotein, and SNCA formation. These results indicate that AETO inhibits DA-induced apoptosis, which is closely related to the suppression of intracellular tyrosinase activity and the formation of α-syn, ROS, and quinoprotein in SH-SY5Y cells [94].

The sulfur-containing compounds derived from garlic have various biological actions. *S*-allylcysteine (SAC, Figure 2D), the most abundant organosulfur compound in aged garlic extracts, has been evaluated for its protective actions against oxidative stress induced by MPP^+^ in the striatum of C57BL/6J mice. Pretreatment with SAC (125 mg/kg i.p.) daily for 17 days, followed by administration of MPP^+^ (0.72 mg/kg i.c.v.), significantly attenuates MPP^+^-induced loss of striatal DA levels (32%). SAC significantly blocks (100% of protection) lipid peroxidation and reduction of superoxide radical production indicated by up-regulation of Cu-Zn-superoxide dismutase activity in MPP^+^-induced mice. Behavioral analyses showed that SAC improves MPP^+^-induced impairment of locomotion (35%). These findings suggest that SAC attenuates MPP^+^-induced neurotoxicity in the striatum of mice through its potent antioxidant effect against oxidative stress induced by MPP^+^ [95].

The neuroprotective role of organoselenides using a differentiated human neuroblastoma SH-SY5Y cell line challenged with 6-OHDA has been investigated [96]. Ebselen is a synthetic organoselenide that mimics the activity of glutathione peroxidase both *in vitro* and *in vivo* [97,98]. Due to its antioxidant function, it can neutralize free radical damage and also has neuroprotective effects against brain injuries involving the glutamatergic system [99]. Ebselen inspired several research groups to synthesize other low-molecular-weight compounds with high availability. In addition, the ebselen analog diphenyl diselenide 2 inhibits glutamate uptake in rat hippocampus [100], and also confers neuroprotection in hippocampus slices through antioxidant mechanisms [101]. Lopes *et al*. (2012) screened several organoselenides investigating their antioxidant potential, and two organoselenides namely, ebselen and diphenyl diselenide (Figure 2E,F), at 3 μM concentrations showed neuroprotective potential in differentiated human neuroblastoma SH-SY5Y cells challenged with 6-OHDA. The authors indicated that these selected organoselenium molecules could be further developed as potential pharmacological and therapeutic drugs to treat PD.

**Figure 3:**
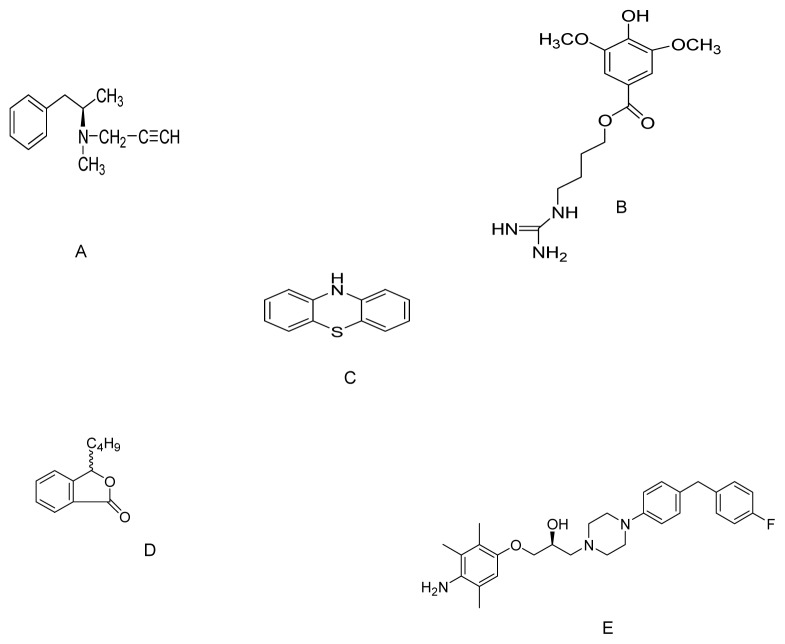
The molecular structure of Deprenyl (**A**); SCM198 (**B**); Phenothiazine (**C**); dl-3*n*-Butylphthalide (**D**) and SUN N8075 (**E**).

Deprenyl is a selective MAO-B inhibitor (Figure 3A) used in clinics to slow the progression of symptoms in patients with PD. Activation of nuclear factor-E2-related factor-2 (Nrf2) has been identified as an alternative mechanism by which deprenyl slows PD progression [102]. Furthermore, chronic treatment with deprenyl induces indirect antioxidant activity by enhancing the expression of antioxidative enzymes such as SOD1, SOD2, and CAT [103]. In a recent study, deprenyl (10, 20, 50 and 100 μM) up-regulated NQO1 expression and activity, attenuated the increase in quinoprotein levels in MPP^+^-treated PC12 cells, and protected against oxidative damage by triggering the Nrf2/ARE pathway. Moreover, its effect on NQO1 upregulation was greatly attenuated in Nrf2 siRNA transfected cells. Activation of Nrf2/ARE signaling by deprenyl in PC12 cells is independent of MAO-B inhibition [104].

SCM198 (4-guanidino-*n*-butyl syringate, Figure 3B) is a chemically synthesized compound that exhibits cardioprotective effects in myocardial infarction models [105] as well as neuroprotective effects on middle cerebral artery occluded rats [106–108]. Shi *et al*. (2011) investigated the neuroprotective effects of SCM198 on 6-OHDA-induced behavioral deficits in rats and cytotoxicity in neuronal SH-SY5Y cells. Pretreatment with SCM198 (0.1, 1, and 10 mM) significantly increased SOD activity, ameliorated intracellular ROS generation, prevented the dissipation of mitochondrial membrane potential, decreased apoptotic cell death, down-regulated Bax, and up-regulated Bcl-2 mRNA and protein levels compared with those in 6-OHDA damaged cells. Intragastric administration of SCM198 at 18 or 60 mg/kg/day for four weeks significantly ameliorated apomorphine-induced contralateral rotations in 6-OHDA-lesioned rats. The authors indicated that the underlying mechanisms of SCM198 for delivering potent neuroprotective effects against 6-OHDA-induced toxicity both *in vivo* and *in vitro* might be by inhibiting oxidative stress and apoptosis [109].

Phenothiazine (Figure 3C) is an organic compound that occurs in various anti-psychotic and antihistaminic drugs. A number of clinically used phenothiazine derivatives, acting as histamine and DA receptor antagonists, exert antioxidant effects *in vitro* [110]. Phenothiazine possesses an exceptionally high antioxidant efficacy against MPP^+^ and rotenone neurotoxicity *in vitro* [111]. Mocko *et al*. (2010) developed the MPP^+^- and rotenone-based *C. elegans* model of DAergic neurotoxicity to perform a systematic analysis of the neuroprotective and behavioral effects of the phenothiazines. They found that 500 nM phenothiazine exerts strong neuroprotective effects at the cellular level and results in better performance on behavioral assays. Thus, chain-breaking agents such as phenothiazine can be developed as therapeutic agents for PD as they rescue DAergic toxicity *in vivo* at nanomolar concentrations based on potent antioxidant properties [112]. Although the doses tested *in vitro* and *in vivo* in PD models are far below the toxic level, side effects such as extrapyramidal symptoms including akathisia and tardive dyskinesia, hyperprolactinaemia, neuroleptic malignant syndrome and as well as substantial weight gain need to be addressed [113,114].

Huang *et al*. (2010) evaluated the therapeutic potential of dl-3n-butylphthalide (NBP, Figure 3D) for treating PD. NBP is safe and currently used in clinical trials for patients with stroke. NBP (0.1, 1.0 and 10 μM) reduces MPP^+^ cytotoxicity by suppressing the mitochondrial permeability transition, reducing oxidative stress, and increasing cellular GSH content in MPP^+^-treated PC12 cells. Moreover, NBP also reduces accumulation of SNCA, the main component of Lewy bodies [115].

Another novel antioxidant, SUN N8075 (Figure 3E), is currently in clinical trials for patients suffering from stroke [116]. Previous studies have revealed a potent neuroprotective activity of this agent in an *in vivo* transient middle cerebral artery occlusion model. The authors suggested that the underlying neuroprotective mechanism might partly involve protection against oxidative stress [117]. The same group investigated the neuroprotective effects of SUNN8075 *in vitro* on both H_2_O_2_-induced ROS production and 6-OHDA-induced cell death in human neuroblastoma SH-SY5Y cells. They also evaluated its putative neuroprotective effects on MPTP-induced neurotoxicity in an *in vivo* mouse model of PD. SUNN8075 treatment at micromolar concentrations significantly decreased the H_2_O_2_-induced production of ROS and protected against 6-OHDA-induced cell death. Intraperitoneal injections of SUNN8075 (30 mg/kg, twice with a 5 h interval) inhibited lipid peroxidation in the mouse forebrain *in vivo*. Moreover, SUN N8075 (10 and 30 mg/kg i.p., twice) exhibited significant protective effects against the MPTP-induced decrease in TH-positive cells in the *substantia nigra*. The authors concluded that the protective effects of SUN N8075 in experimental PD models was, at least in part, via an anti-oxidation mechanism [118].

**Figure 4:**
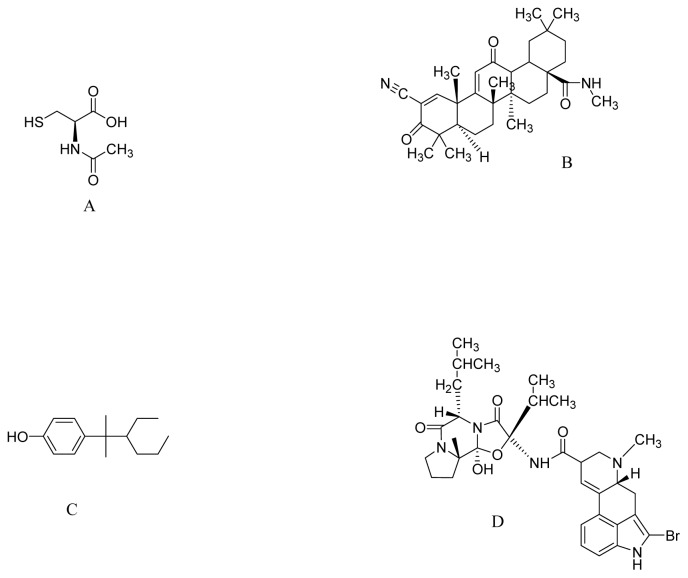
The molecular structure of *N*-acetyl-l-cysteine (**A**); CDDO-methyl amide (**B**); NP7 (**C**); Bromocriptine (**D**).

*N*-acetyl-l-cysteine (NAC, Figure 4A), is a pharmaceutical drug and nutritional supplement used primarily as a mucolytic agent and in the management of paracetamol overdose. Recently, Clark *et al*. (2010) hypothesized that NAC supplementation in drinking water (40 mM) protects against SNCA toxicity. Oxidative stress may increase the accumulation of toxic forms of SNCA in a DA-dependent manner [119]. Transgenic mice over-expressing wild-type human SNCA drank water supplemented with NAC from ages six weeks to one year. As a result, NAC increased SN levels of GSH within five to seven weeks of treatment. The authors found that the loss of DAergic terminals at one year associated with SNCA over-expression was significantly attenuated by NAC supplementation. Furthermore, NAC significantly decreased the levels of human SNCA in the brains of PDGFb-SNCA transgenic mice compared to those in controls. The authors suggested that increased oxidative stress due to early GSH deficiency in the SN may lead to enhanced toxicity of SNCA in DAergic SN neurons, suggesting that strategies to increase GSH or to block oxidative stress by NAC may protect against the SNCA toxicity seen in PD [120].

Oleanolic acid is a triterpenoid, which has been used for centuries in Asian medicine, due to its anti-inflammatory activity. Synthetic triterpenoids are potent inducers of Nrf2 transcriptional activity, resulting in marked induction of NADPH quinone oxidoreductase-1 (NQO-1), hemeoxygenase-1, GSH transferases, and other cytoprotective enzymes. Yang *et al*. (2009) investigated the synthetic triterpenoid, CDDO-methyl amide (2-cyano-*N*-methyl-3,12-dioxooleana-1,9(11)-dien-28 amide; CDDO-MA) (Figure 4B), which is at least 200,000 times more potent than its naturally occurring distant parent, oleanolic acid, as an inducer of NQO-1. CDDO-MA (800 mg/kg of diet) exerted profound neuroprotective effects against MPTP and 3-nitropropionic acid neurotoxicity. The authors showed that the neuroprotective effects were due to its antioxidant effects, caused by induction of pathways known to be regulated through the Nrf2/antioxidant response element (ARE) signaling pathway, such as GSH synthesis [121].

NP7 (Figure 4C) is a new marine derived antioxidant, with a different chemical molecular structure than that of classic phytochemicals, obtained by lead optimization system from *Streptomyces* spp. NP7 has profound free radical scavenging properties in the sub μM–nM range and easily crosses the blood brain barrier of mammals [122]. Meena *et al*. (2009) studied the protective effects of NP7 on cell death induced by oxidative stress in neuronal and glial midbrain cultures from parkin null mice (PK-KO). NP7 (5–10 μM) prevented H_2_O_2_-induced apoptosis and necrosis of midbrain neuronal and glial cultures from wild type and PK-KO mice. NP7 suppressed microglial activation and the H_2_O_2_-induced dropout of DA neurons. The authors indicated that NP7 might be a promising neuroprotecting agent against oxidative stress in PD [123].

Bromocriptine, a DA agonist (Figure 4D) has been widely used in PD clinics since 1974 to delay and minimize deleterious motor fluctuations after long-term l-dopa treatment [124]. In addition to its action as a DA receptor agonist, it reduces the formation of oxygen radicals during the course of normal levodopa and DA metabolism [125]. Recent observations suggest that bromocriptine is a free radical scavenger that scavenges hydroxyl and superoxide radicals *in vitro* and acts as an antioxidant that inhibits free radical formation [126]. Based on these properties, Lim *et al*. (2008) studied the cytoprotective mechanism of bromocriptine against oxidative damage in H_2_O_2_-treated PC12 cells. Bromocriptine (5 μM) up-regulated the expression and activity of the antioxidant enzyme NQO1, attenuated the increase in the protein-bound quinone in H_2_O_2_-treated PC12 cells, protected PC12 cells against oxidative damage, and increased the expression and nuclear translocation of Nrf2. The Nrf2-related cytoprotective and antioxidative effects of bromocriptine are independent of DA receptor activation [127].

Several other synthetic compounds such as selenium [128], *R*-alpha-lipoic acid [129], rosmarinic acid [130], eugenol [131] isoborneol [132], melatonin [133], metalloporphryins compounds, and metal ion chelators [134] have neuroprotective effects in PD models based on their antioxidative properties. The antioxidant synthetic compounds reviewed are summarized and represented in Table 1.

## 5. Conclusions

Despite the availability of many drug classes such as l-dopa, DA agonists, monoamine oxidase inhibitors, catechol-O-methyltransferase inhibitors and anticholinergic agents for the symptomatic treatment of PD, a cure remains elusive. Although the exact nature of mechanism that involves neurodegeneration in PD is not well understood, oxidative stress is one of the major risk factors that could initiate and/or promote degeneration of DA neurons. Therefore, antioxidant therapy could prevent or reduce the rate of progression of this disease. It has been demonstrated that antioxidant compounds are able to protect neuronal cells by scavenging free radicals or activating the antioxidant mechanisms. Numerous *in vitro* and *in vivo* animal studies reported during the last five years centered on oxidative stress and ROS mediated mechanisms such as radical scavenging, metal chelating and/or regulation of antioxidant enzymes. However experimental evidence also showed that oxidative stress is not the sole deleterious factor implicated in the death of DAergic neurons and other mechanisms involving modulatory effects on signal transduction pathways and gene expression may also play key roles in the neuroprotection of progressive PD. The compounds discussed in this review may also act by regulating these pathways along with antioxidative mechanisms, which may be synergistic for delivering beneficial effects in PD. In addition, combination therapy with antioxidants and existing drugs might also be beneficial and enhance the efficacy of standard therapy in the treatment of PD. Various types of free radicals are produced, and antioxidants vary in their ability to quench these different free radicals, therefore supplementation with multiple antioxidants and relying on a cocktail of agents, each specifically targeting one aspect of the degenerative mechanism in the correct time frame and dose, may provide better results to achieve promising clinical effects. However, examining the critical factors including the optimum concentrations required, what biologically active forms are needed and crossing of these agents into blood brain barrier to exert potential therapeutic benefits are indeed essential. A complete understanding of the molecular mechanisms of the ROS specificities in PD, and larger studies, both epidemiologic and randomized clinical trials in humans, as well as animal studies, are urgently needed to confirm these findings for delivering beneficial effects in the treatment of PD.

## Figures and Tables

**Table 1:** Summary of recent antioxidant compounds exhibiting neuroprotection in experimental models of PD.

Compound	Model	Effective dose	Antioxidant activity	Ref.
Curcumin	MPTP mouse model	Dietary supplementation	Increase in GSH levels and protected against peroxynitrite-mediated inhibition of brain mitochondrial complex I.	[57]
	A53T SNCA-induced toxicity in PC12 cells	0.1, 0.5 and 1.0 μM	Decrease in oxidative stress and apoptosis	[58]

Quercetin	6-OHDA-induced toxicity to PC12 cell	25, 50 and 100 μM	Suppression of oxidative stress.	[54]
	6-OHDA-induced toxicity to zebrafish	6 and 12 μM	Protect against 6-OHDA-induced apoptosis. Decrease in dopaminergic neuron loss.	[65]

Coenzyme Q10	Acute MPTP model	1600 mg/kg/day	Protection against dopamine loss.	[75]
	Chronic MPTP model	1600 mg/kg/day, via diet	Increase in CoQ10 plasma concentration	

Creatine with CoQ10	MPTP mouse model	2% Creatine & 1% CoQ10 in diet	Reduced lipid peroxidation and alpha-synuclein accumulation.	[77]

Resveratrol	6-OHDA model	20 mg/kg per day	Decrease in ROS. Increase in antioxidant capability of nigral tissues.	[82]

Luteolin	ROS insult to neural cells	5, 10, and 20 μM	Decrease in ROS production and increase the activities of catalase and glutathione.	[85]

Idebenone	HtrA2 knockout mice	500 mg/kg body weight/day orally	Extends lifespan and improves motor symptoms. Regulation of apoptotic pathway	[93]

AETO	SH-SY5Y cells	0.4, 2, and 10 μM	Suppression of ROS & DA-induced apoptosis	[94]

*S*-allylcysteine	MPTP mouse Model	125 mg/kg; i.p.	Blocks lipid peroxidation and reduction of superoxide production	[95]

Ebselen & Diphenyl diselenide	6-OHDA-induced toxicity to SH-SY5Y cell	3 μM each	Peroxyl radical scavenging. Increase the GPx activity and SOD activity.	[96]

Deprenyl	MPP^+^ treated PC12 cells	10, 20, 50 and 100 μM	Nrf2/ARE pathway	[104]

SCM198	6-OHDA-induced toxicity to SH-SY5Y cells	0.1, 1, and 10 mM	Increase in SOD activity. Suppression of apoptosis	[109]

Phenothiazine	MPP^+^ and rotenone toxicity to *C. elegans*	500 nM	Increase free radical scavenging effects	[112]

dl-3*n*-Butylphthalide	MPP^+^ treated PC12 cells	0.1, 1.0 and 10 μM	Reducing oxidative stress & increasing cellular GSH content	[115]

SUN N8075	MPTP mouse model	10 and 30 mg/kg i.p	Inhibited lipid peroxidation and H_2_O_2_-induced ROS.	[118]

*N*-acetyl-l-cysteine	Transgenic mice overexpressing α-synuclein	Drinking water supplemented with 40 mM	Increase of GSH levels in SN.	[120]

CDDO-methyl amide	MPTP and 3-nitropropionic acid induced neurotoxicity	800 mg/kg of diet	Nrf2/ARE pathway	[77]

NP7	Parkin null mice	5–10 μM	Inhibits H_2_O_2_-induced apoptosis	[123]

Bromocriptine	H_2_O_2_-treated PC12 cells	5 μM	Increase activity of NQO1 and Nrf2 signaling	[127]
